# Semi-Supervised Framework with Autoencoder-Based Neural Networks for Fault Prognosis

**DOI:** 10.3390/s22249738

**Published:** 2022-12-12

**Authors:** Tiago Gaspar da Rosa, Arthur Henrique de Andrade Melani, Fabio Henrique Pereira, Fabio Norikazu Kashiwagi, Gilberto Francisco Martha de Souza, Gisele Maria De Oliveira Salles

**Affiliations:** 1 Department of Mechatronics and Mechanical Systems Engineering, Polytechnic School, University of São Paulo, São Paulo 05508-010, SP, Brazil; 2 Informatics and Knowledge Management Graduate Program, Universidade Nove de Julho, São Paulo 01525-000, SP, Brazil; 3 Companhia Paranaense de Energia—COPEL, Curitiba 80420-170, SP, Brazil

**Keywords:** deep neural networks, fault prognosis, autoencoder, remaining useful life (RUL)

## Abstract

This paper presents a generic framework for fault prognosis using autoencoder-based deep learning methods. The proposed approach relies upon a semi-supervised extrapolation of autoencoder reconstruction errors, which can deal with the unbalanced proportion between faulty and non-faulty data in an industrial context to improve systems’ safety and reliability. In contrast to supervised methods, the approach requires less manual data labeling and can find previously unknown patterns in data. The technique focuses on detecting and isolating possible measurement divergences and tracking their growth to signalize a fault’s occurrence while individually evaluating each monitored variable to provide fault detection and prognosis. Additionally, the paper also provides an appropriate set of metrics to measure the accuracy of the models, which is a common disadvantage of unsupervised methods due to the lack of predefined answers during training. Computational results using the Commercial Modular Aero Propulsion System Simulation (CMAPSS) monitoring data show the effectiveness of the proposed framework.

## 1. Introduction

Incorporating IoT into maintenance has brought new possibilities, including condition-based maintenance (CBM). CBM aims to avoid unnecessary maintenance tasks by taking maintenance actions only when there is evidence of abnormal behavior of physical assets [1,2]. If a CBM program is appropriately established and effectively implemented, it can significantly reduce maintenance costs by reducing the number of scheduled preventive maintenance operations [3].

The main feature of CBM is the condition monitoring (CM) process, in which signals are continuously monitored from certain types of sensors or other appropriate indicators to show the current state of a system or component [4]. Thus, a CBM program consists of three key steps [4]: data acquisition (information collection), data processing (information understanding and interpretation), and decision making (aimed at recommending efficient maintenance policies).

The sequence of steps mentioned above results in two essential forms of analysis within a CBM program: fault diagnosis and prognosis. While diagnosis deals with fault detection and isolation of faulty components, prognosis aims at predicting when the diagnosed fault will turn into a failure, i.e., its goal is to estimate how soon and how likely this failure is to occur [3]. A comprehensive description of prognosis and prognosis modeling is provided by ISO 13381-1 [5], which defines it as “an estimate of the time to failure and risk for one or more existing or future failure modes.” Such an estimate is often referred to as remaining useful life (RUL).

Deep learning (DL) methods have recently been gaining ground in prognosis and health management as they are solutions capable of identifying and predicting the equipment condition through large datasets. They are helpful in circumstances where there is little or no investigation into the physics of the failure. However, the current state of the art concerning industrial-scale integrated solutions is still incipient since many works use simulated databases or real data with artificial faults. Thus, some challenges must be surpassed, such as the ability to learn in environments with evolving operating conditions, novelty detection, robustness to changes in the operational conditions, the capacity of generalization, and output interpretability [6].

Deep learning embraces neural network learning models with multiple layers of computational units that are capable of decomposing higher-level abstract features in terms of other more straightforward representations [7]. As an extension of the single-layer networks, it is also suitable for supervised, unsupervised, and semi-supervised types of learning.

DL emerges in prognostics and health management (PHM) as a resource to solve previously intractable problems in the field, improve performance over traditional techniques, and reduce the effort to deploy prognostic systems due to its advantages. Fink et al. [6] listed some of them as the ability to automate the processing of a significant amount of condition monitoring data, extract valuable features from high dimensional, heterogeneous data sources, learn functional and temporal relationships between and within the signal time series, and transfer knowledge between different operating conditions and different units. Furthermore, DL contributes to attenuating the need for feature engineering in datasets composed of many monitored variables, which is demanding, by incorporating it inside its own network [6].

Several studies have applied DL techniques to solve fault diagnosis and prognosis problems. Tao et al. [8], for example, studied the different structures of a two-layer network designed by varying the hidden layer size and evaluated for its impact on fault diagnosis.

Babu et al. [9] built a convolutional neural network (CNN) to predict the RUL of a system using the readings of several sensors as the input. The authors conducted a series of experiments and demonstrated how a CNN-based regression model could outperform three other regression methods, i.e., the multilayer perceptron, the support vector regression, and the relevance vector regression.

Among the authors that explored the signal reconstruction approach, Malhotra et al. [10] combined LSTM layers in an encoder-decoder to attain an unsupervised health index for a system using multi-sensor time-series data. The study concludes that LSTM-ED constructed HI learned in an unsupervised manner can capture the degradation in a system and that this HI can be used to learn a model for RUL estimation with equivalent performance to domain knowledge or exponential and linear degradation model assumptions.

Wu et al. [11] have proposed a semi-supervised diagnosis architecture called “hybrid classification autoencoder,” which uses a softmax layer over the encoded features of the autoencoder. In its approach, vibration data are pre-processed in a bi-dimensional entry by a short-time Fourier transform (STFT) and subjected to consecutive convolutional layers. Experimental validation has been performed in a publicly available dataset of moto-bearing signals. The authors also presented a practical application in a hydro generator rotor diagnosed with a rub-impact fault between the turbine shaft and turbine guide bearing.

Moreover, several diagnosis and fault prognosis models and frameworks are available in the literature for the most diverse scenarios. One of the first was elaborated by Vachtsevanos et al. [12], and it divides the process into seven steps, starting from sensor data collection, passing through FMEA analysis, operating mode identification routine, feature extractor, and sequential diagnostic and prognostic modules.

Other researchers [13] prefer explicitly declaring the health index (HI) construction as a step of the prognostic scheme and prefer discerning the health stage (HS) of the system by the indicator. The health stage division in the Lei et al. [13] framework shares similarity with the fault detection and diagnostic actions but has the particular goal of splitting a degradation pattern into different ¨health stages¨ according to variations in its characteristics.

Even some standards attempt to generalize a conceptual framework aiming to provide basic CBM and PHM modules from data acquisition and analysis to health assessment, prognostic assessment, and advisory generation. ISO 13381-1 divides this process into four actions (one preprocessing and three prognostic types or levels of increasing complexity): data preprocessing, existing failure mode prognosis, future failure mode prognosis, and post-action prognosis.

However, although many authors conclude that semi-supervised or unsupervised learning-based methods embedded in frameworks are more appropriate for multiple reasons (see [14], for example), there is still a predominance of supervised approaches that depend on intensive manual intervention to label the data. Additionally, supervised methods cannot find previously unknown patterns in data, which are not rare in industrial environments where various causes for the failures result in very different behaviors of each signal before different incidents, even of the same type [14]. According to Sikorka et al. [15], most of the research on prognosis has been theoretical and restricted to a small number of models and failure modes. There are few published examples of prediction models applied in complex systems exposed to various operational and business conditions.

So, the main objective of this work is to provide a semi-supervised framework based on autoencoder deep learning methods for fault detection and prognosis. To overcome a common limitation of unsupervised methods related to the lack of predefined answers during training, this work provides a set of metrics designed to measure the accuracy or effectiveness of the models, ensuring comparability between them for validation and improvement purposes.

Thus, this work not only expands the literature on semi-supervised methods for fault prognosis but also provides a generic framework based on an autoencoder deep learning method. Consequently, the contributions of the proposed approach can be stated as follows:

1. This approach provides a systematic framework for implementing a semi-supervised prognosis method based upon an autoencoder deep learning method;
2. This approach implements a framework designed for application in the industrial scenario since it considers the system’s restrictions such as data management, the physical behavior of degradation processes, and business specifications;
3. This approach enables the detection of different kinds of faults by evaluating each sensor channel (i.e., variable) individually;
4. This approach proposes a set of metrics to evaluate the accuracy and effectiveness of the fault detection and prognosis models.

The effectiveness of the proposed framework is tested using the Commercial Modular Aero Propulsion System Simulation (CMAPSS) for monitoring data, which is largely used for testing fault detection, diagnosis, and prognosis [16,17,18,19,20,21,22].

The rest of this text is structured in the following way: Section 2 presents the proposed framework in detail; Section 3 shows the results obtained by applying the framework to the CMAPSS database; and Section 4 presents the conclusions and discussions arising from this work.

## 2. The Proposed Framework

The proposed approach relies upon generating a prognosis horizon for fault degradation patterns using the reconstruction error extrapolation of a deep autoencoder trained only with the machine’s normal operating condition monitoring data. This work focuses on detecting and isolating possible divergences in the monitoring measurements, which may indicate a fault, and extrapolating their growth to predict the machine’s RUL. Such extrapolation is conducted using a set of more straightforward univariate functions with known behavior until a limit of divergence, signaling the failure’s occurrence.

The reason for using this approach, in contrast with what is recently adopted in the prognosis literature, is that there is a demand for models capable of following, recording, and interpreting machine behavior in the context of complex engineering systems (CES). Currently, industrial equipment is generally assisted by monitoring systems (which are either automated or controlled by humans). These systems are usually assisted by programmed fault alarms based on guidelines or empirical knowledge about the process, composing the resources for predictive maintenance. This type of setup is classified in Level 0 in terms of prognostic implementation readiness according to ISO 13381 classification [5], i.e., they are CES with monitoring infrastructure capable of performing detection and sometimes fault identification, yet they do not form a strong foundation for most sophisticated prognostic techniques that require intensive and systematical diagnostic capacities.

In line with the above, the proposed model constitutes a framework that has the potential to embrace all the past requirements—which are the detection, isolation, and identification of the fault—for the remaining useful life prediction reaching Level 1 prognostics according to the same standard. In fact, since the autoencoder is a signal reconstructor and therefore can work as a hidden state reconstructor, diagnosis is possible because each channel could be compared individually to provide a multilabel classification of different kinds of faults.

**Figure 1:**
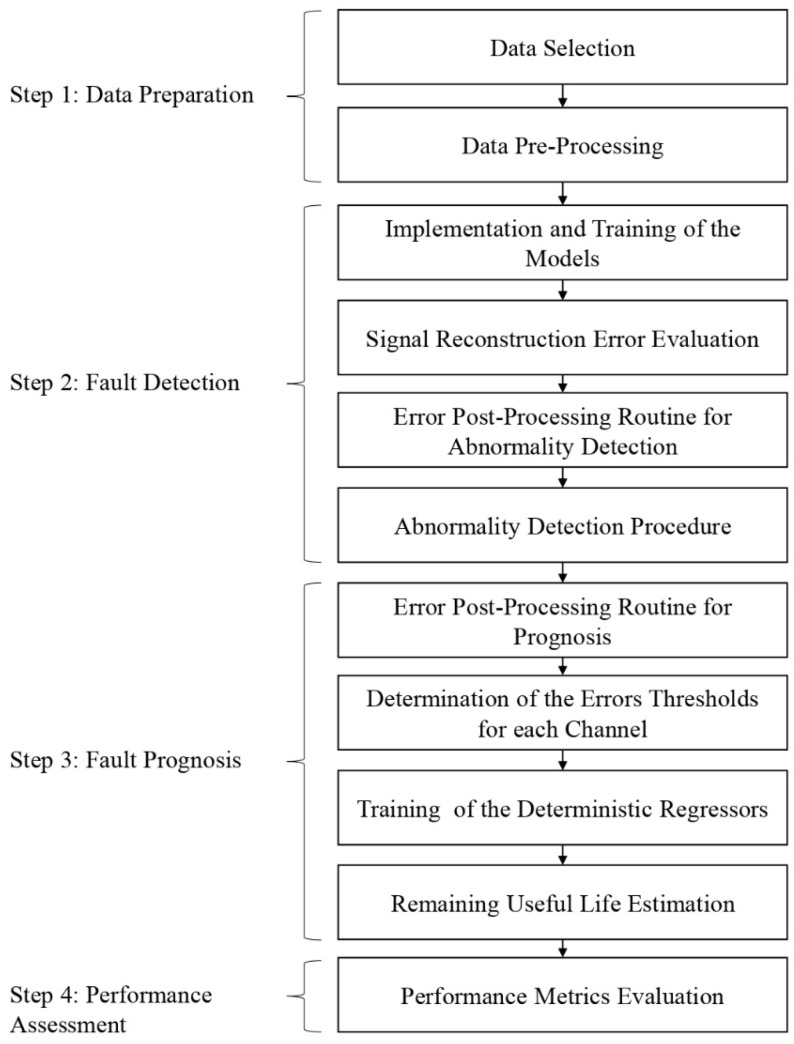
Flowchart describing the prognostic framework.

Another reason for adopting this approach is the unbalanced proportion of monitoring data between faulty and non-faulty conditions in an industrial scenario since some failure events are rare for certain types of equipment. Then, the availability of a great array of data in the normal operating conditions of a CES is attractive for the use of data-driven methodologies. The steps for implementing the prognosis framework and RUL prediction are expressed in the flowchart in Figure 1.

### 2.1. Step 1: Data Preparation

The data selection comprises the procedure of selecting data in normal operational conditions (NOC), and it is necessary to characterize this state beforehand with the help of a specialist or some reference criteria (for example, collecting data immediately after maintenance or an arbitrary time interval before the occurrence of a fault). It is interesting pointing out that there is no need to establish a perfect boundary in the transition of the conditions since it is desired to detect incremental abnormalities.

After that, the data are scaled using the given criteria, which could be achieved by using a value range reference or by removing the mean and scaling to unit variance—standard score—according to the dataset profile in the application example. It is worth emphasizing that only NOC-labeled data are applied to calibrate the scaler to avoid distortions in the set designated to train the networks. Following this, the data are reshaped into a set of subsequences of size *n* that will supply the models. These subsequences are generated through a moving window with a temporal iteration step of *p*, thus allowing overlapping of *n–p* samples. Each sample has shape (*n, m*) (*n* is the subsequence size, and *m* is the number of channels—sensors inputs). For this work, NOC-labeled data are split between train and validation sets to prepare the neural network. The terminology test set will be designated exclusively to refer to data not applied in the DNN training and tunning process, including abnormal-labeled data.

### 2.2. Step 2: Fault Detection

**Figure 2:**
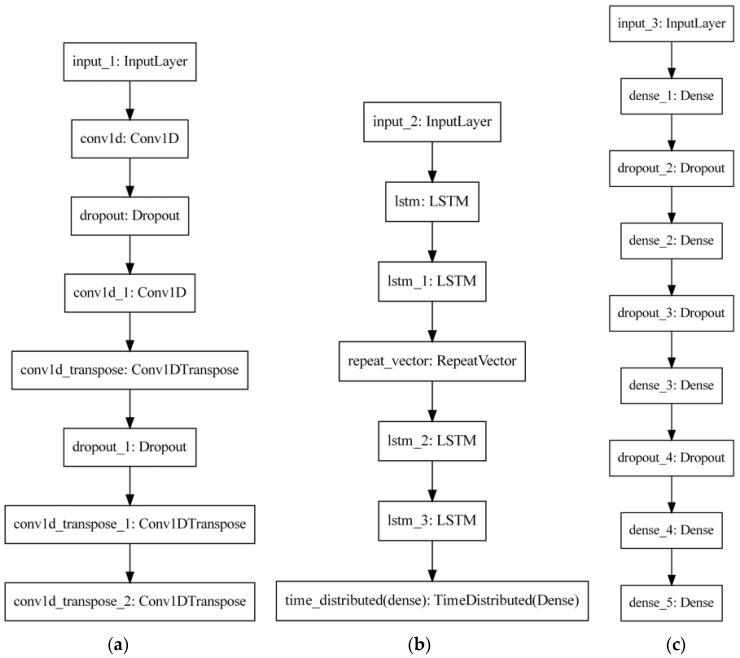
Representation of the autoencoders’ architectures. Each box indicates a layer, and the arrows indicate the information flow. (**a**) Conv-1d. (**b**) LSTM. (**c**) MLP.

The DNN autoencoder models are programmed according to the hyperparameter specifications in Table 1, and their structures are illustrated in Figure 2. Three different kinds of layers will be used, namely MLP, LSTM, and 1D-Convolutional (1D-CNN or Conv-1d), which are commonly used in the setup of DL models to monitor signals in the literature. Albeit MLP could express poor performance in comparison with the other layers, it is applied in this study as a reference to analyze the models. Thus, a minimum requirement for them is to outperform a classic MLP perceptron architecture. Recurrent neural networks, especially LSTM, are widely used for PHM applications. Moreover, Conv-1d is an alternative to apply convolutional operations into time-series data without demanding transformations to the bi-dimensional spatial space, which is time-consuming. In addition, Conv-1d is less computationally expensive because it has fewer parameters.

Rosa et al. [23] investigated the sensitivity of AE architecture hyperparameters over its abnormality detection performance. The study concluded that some specific hyperparameters influence the model outcomes more than others, therefore serving as a reference for the search space definition. Although easy to implement, grid search is an exhaustive procedure that is inefficient without prior knowledge of the search space near the optimality. Some alternatives are the random search or search based on the Bayesian optimization theory [24].

The reconstructed signal subsequences outputted from the trained AE models are compared with the actual signal observations, and the reconstruction error (*RE*) is evaluated. *RE* is computed as a mean squared error (MSE) function applied in a subsequence for each channel, so it is possible to inspect discrepancies individually. The subsequences are addressed by the time index of the last observation; then, the *RE* is also assigned for this position. Thus, the reconstruction error follows the notation below:

(1) $$RE_{i,j}=\frac{\sqrt{\left|s_{i,j}-s_{i,j}^{r}\right|}}{n}$$

where *RE_i_*_,*j*_ is the reconstruction error for the subsequence $s_{i,j}=\left\{s_{i,j}^{\left(0\right)},\ldots ,s_{i,j}^{\left(n\right)}\right\}$ with temporal index *i*, channel index *j* and size *n*. $s_{i,j}^{r}$ correspond to the reconstructed subsequence mimicking *s_i_*_,*j*_.

The abnormality detection procedure comes afterward, employing the reconstruction error matrix, *RE*, whose entries are defined by Equation (1), to build a set of error threshold functions *f_th_*(*t*) that is used to classify whether or not a data entry is abnormal. First, it is important to note that the *RE_1_*_:*n*,*j*_ series are subject to local variability due to the outliers that could come from the sensor’s readings. For example, in machines with more than one operational mode or those with intermittent operations, the working routine is cyclical, having unstable behaviors during state transitions or due to variations in the cycle periods. Examples are the take-off and landing of aircraft or the switch between generation and motorization modes in hydro-generators. Moreover, it is not a trivial task to characterize state transitions, even for experts in the process, and it is part of the data selection step of this study. This fluctuation could severely affect the method’s abnormality detection capacity and must be considered in relation to the definition of *f_th_(t)* and interpretation of the entries of *RE*.

**Figure 3:**
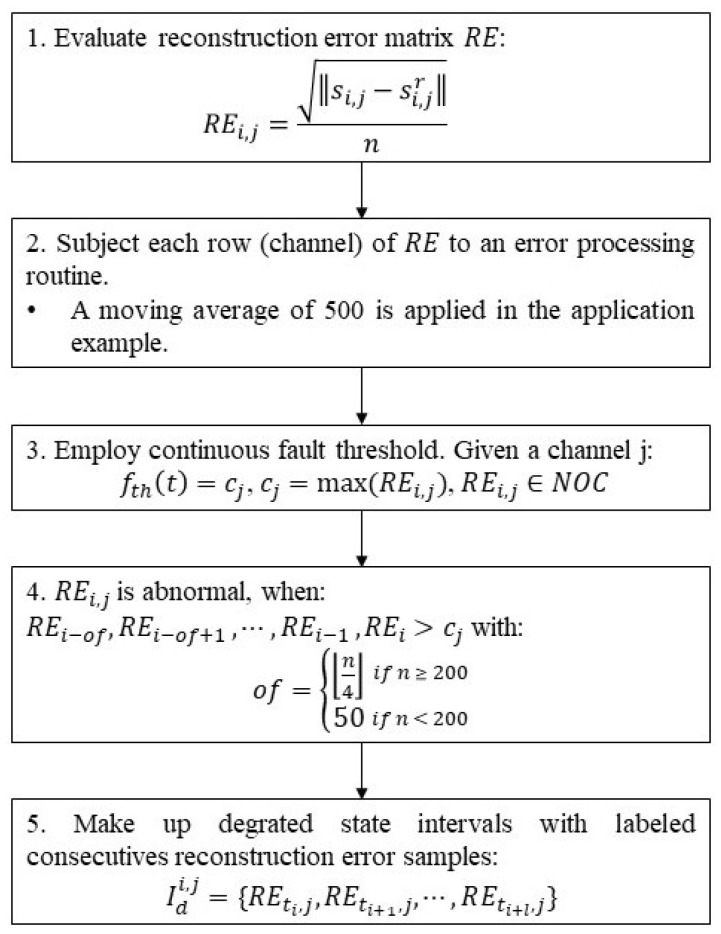
Flowchart systematizing the abnormality detection procedure.

**Figure 4:**
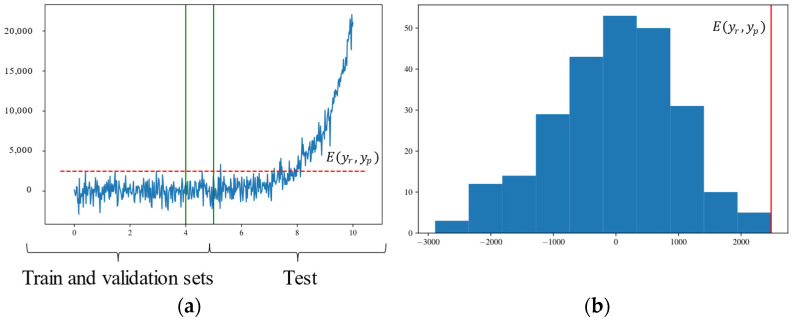
Exemplification of the procedure in Step 2. In (**a**), there is a temporal progression of the reconstruction errors for an arbitrary variable. Train—from zero to the first green line—and validation—interposed between the green lines—sets correspond to NOC, while the test set is in the degraded condition. *E*(*y_r_*, *y_p_*)—dashed red line—is the maximum error between the samples in the condition labeled as normal. The localization of *E* in the samples’ distribution is displayed in (**b**) as a continuous red line. As the monotonic pattern evolves, it exceeds *E*, and if a certain quantity of consecutive *RE* keeps above the limit, the abnormality is registered.

The main part of Step 2 is summarized in the flowchart presented in Figure 3 and exemplified by the graphics in Figure 4. The non-conformities are detected in this work by using a set of continuous threshold functions *f_th_*(*t*) = *c_j_*, (*c_j_* is the maximum value between the post-processed *RE* samples labeled as NOC for the *j*th channel). Samples of the post-processed *RE_1_*_:*n*,*j*_ that exceed *c_j_* are labeled as abnormalities. Sometimes the post-processing of *RE* alone is not enough to avoid the occurrence of false positives, which are caused by pointwise or small cluster point addressing. To highlight the cumulative abnormality resulting from the monotonic growth of the degradation pattern, an offset of consecutive abnormal labeled points is used as a requirement for pointing out the beginning of the degradation.

It is worth mentioning that several other techniques can be used in each step of the proposed framework. Specifically, regarding fault detection, the objective of the proposed method is similar to that of the multivariate statistical process control (MSPC). However, despite the usefulness of MSPC for multivariate surveillance in industrial practice, there are some disadvantages regarding establishing what happened in the process. The need for a mathematical background is another drawback for applying MSPC in real scenarios [25].

### 2.3. Step 3: Fault Prognosis

The RUL estimation is developed from the samples inside the degraded state intervals (*I_d_*) provided by the abnormality detection procedure. *I_d_* is defined as a set of consecutive reconstruction error samples, meaning that:

(2) $$I_{d}^{i,j}=\left\{RE_{t_{i},j},RE_{t_{i+1},j},\cdots ,RE_{t_{i+l},j}\right\}$$

where $I_{d}^{i,j}$ is the *i*th interval with cardinality *l* + 1 for the channel *j*, {*t_i_*, *t_i+_*_1_, ⋯, *t_i+l_*} is the ordered set of temporal indexes addressed for the *RE*s. An additional notation is $I_{d}^{t_{d},i,j}$, which emphasizes the temporal placement of *I_d_*, which is agreed to be the time of the first abnormal sample within *I_d_*. For convenience, the positional argument could be suppressed occasionally so: $I_{d}^{i,j}\equiv I_{d}^{t_{d},i,j}\equiv I_{d}^{t_{d},j}$. Moreover, if $I_{d}^{\left(j\right)}$ represents the superset of all *I_d_*’s in the channel j, then $I_{d}^{\left(j\right)}⊃I_{d}^{\left(i,j\right)}\forall i$, and to symbolize the union of sets more conveniently, the notation $I_{d}$ is employed, such that $I_{d}=\cup_{j=0}^{m}\cup_{i=1}^{n}I_{d}^{\left(i,j\right)}$ and $I_{d}^{\left(j\right)}=\cup_{i=1}^{n}I_{d}^{\left(i,j\right)}$.

These samples could either be subjected to another post-processing routine specially designed for prognosis or the same routine already made for abnormality detection. Hereupon, the initial goal for RUL evaluation is to determine the prognostic error threshold for each channel, which is equivalent to the failure limit of a built health index or measured quantity. As the RE cannot directly relate to future variations on the input channels—unless explicability techniques are coupled to the DNN—it is necessary to take past failure events as references to determine those thresholds. Thus, the prognostic error threshold $PE_{th}^{\left(j\right)}$ for the *j*th channel is given as an average of *k* reconstructed error samples and *n* fault observations before the failure.

The next step is iteratively fitting curves and executing extrapolations from the first detected abnormality until the error threshold for each channel to obtain the RUL prediction at the time *t*. At the instant *t*, there can be more than one estimation because the degradation evolution of each channel is treated independently and fitted in a univariate function. Therefore, a decision criterion is required to provide a singularized prediction, which is achieved by observing curve fitting metrics, prognostic threshold variability, and the monotonicity of the generated profiles together with the values of the produced estimations. The pseudocode in the Algorithm 1 systematizes details concerning the step above.
**Algorithm 1** Estimation of the RUL for an experimental fault event with the made assumption that the real remaining life is known for study purposes.1:$t_{\mathrm{init}\;}\leftarrow \min\left(t_{d}\left(I_{d}^{\left(0,j\right)}\right):0\leq j\leq m\right)$2:**for**$t_{i}=t_{\mathrm{init}\;}$$\;\mathrm{to}\;t_{\mathrm{eol}\;}$**do**3:
   **for**
j=0 to m
**do**4:

   **if**
$\exists I_{d}^{\left(t_{d}:t_{f},j\right)}$$\;\mathrm{such}\;\mathrm{that}\;RE^{\left(t_{i},j\right)}\in I_{d}^{\left(t_{d}:t_{f},j\right)}$
**and**
$t_{d}\leq t_{i}\leq t_{f}$
**then**5:      $\;\;\;x\leftarrow t_{d}:t_{i}$6:      $\;\;\;y\leftarrow I_{d}^{\left(t_{d}:t_{i},j\right)}$7:            **for**
i=0$\;\mathrm{to}\;n\left(f\right)$
**do**8:        $\;\;\;\;\;\;c^{\left(i\right)}\leftarrow$$\;\mathrm{Solve}:\;\mathrm{Leasts}\;\mathrm{squares}\;\mathrm{to}\;f^{\left(i\right)}$$\;\mathrm{in}\;\left(x,y\right)$9:



$\;\;\;\;\;\;t_{\mathrm{eol}\;}^{*}\leftarrow$$\;\mathrm{Solve}:\;f^{\left(i\right)}\left(c^{\left(i\right)},x\right)-PE_{th}^{\left(j\right)}=0$10:



$\;\;\;\;\;\;r^{*\left(i,j\right)}\leftarrow t_{\mathrm{eol}\;}^{*}-t_{i}$11:


      
**end for**
12:


$\;\;\;\;i^{*}\leftarrow D_{f1}\left(R^{2}\left(f^{\left(i\right)}\left(c^{\left(i\right)},x\right),y\right):0\leq i\leq n\left(f\right)\right)$13:


$\;\;\;\;r^{*\left(j\right)}\leftarrow r^{*\left(i^{*},j\right)}$14:


$\;\;\;\left.Mon^{\left(j\right)}\leftarrow {\mathrm{Mon}}^{\left(f^{\left(i*\right)}\right)}\left(c^{\left(i^{*}\right)},x\right),y\right)$15:


$\;\;\;\;R^{\left(j\right)}\leftarrow R^{2}\left(f^{\left(i^{*}\right)}\left(c^{\left(i^{*}\right)},x\right),y\right)$16:


   **end if**
17:
**end for**18:
$r^{*\left(t_{i}\right)}\leftarrow D_{f2}\left(Mon^{\left(j\right)},R^{\left(j\right)},\sigma \left(PE_{th}^{\left(j\right)}\right),r^{*\left(j\right)}:0\leq j\leq m\right)$19:**end for**

The Algorithm 1 has three main loops that permeate the prognosis procedure in a given inspection interval as long as an abnormality is detected. The loops, from the most to the least nested, iterate through curves (Loop 1), channels (Loop 2), and in time (Loop 3), respectively. The first one adjusts the function shape for a channel m at time $t_{i}$ using the least squares method and estimating the $t_{eol}$ and thus the RUL. The second one, in turn, evaluates the RUL and curve-fitting metrics for each one of the channels with degradation labeled in $t_{i}$ using the decision function $Df_{1}$. By the end, the third loop decides whether a prediction is made at the time $t_{i}$ and its value using the decision function $Df_{2}$. Decision functions are subroutines that hierarchically dispose of the most likely remaining life prediction(s) after inputting a list of them by means of the analysis of a set of curve-fitting metrics. $Df_{1}$ only employs $R^{2}$ in the sorting and eliminates nonsensical outcomes and those below an established fitting limit. The final result comes from the mean of the remnants’ occurrences. $Df_{2}$ considers monotonicity beyond $R^{2}$ and weights the last one in a fraction of 0.8 out of 1.

Furthermore, the curve fitting is executed using a non-linear least-squares problem with bounds on the variables. The objective is to find a local minimum of the cost function *F*(*x*), which is:

(3) $$Minimize\;F(c)=\overset{N}{\underset{i=1}{\sum }}\rho ({q_{i}(c)}^{2}),\;\;Subject\;to:\;x\geq 0$$

where *c* is a vector of estimable parameters, *N* is the number of available data points, $\rho \left(s\right)$ is a scalar loss function that reduces the outliers’ influence, and *q_i_*(*c*) is the component *i* of the vector of residuals q. Residuals are understood as the difference between the prediction of a model function *f*(*x,c*) and a set of data points $\left\{i=1,\ldots ,N\right\}$ so $\;q_{i}\left(c\right)=f\left(x_{i},c\right)-y_{i}$.

Minimization is performed through the trust-region reflective algorithm implemented in an open-source scientific computing library. The used curves are disposed of in Table 2 and represent common degradation patterns found in mechanical components [13,26].

The value of the function $f^{-1}\left(RE\right)$, which is the inverse of $f\left(t\right)$, at the point $p=PE_{th}^{\left(j\right)}$ gives the component $t_{eol}^{*\left(j\right)}$. Thus, the estimated RUL at the instant $t_{i}$ is $r\left(t_{i}\right)=t_{eol}^{*\left(j\right)}-t_{i}$ for the fitted curve.

### 2.4. Step 4: Performance Assessment

The performance assessment is realized with dedicated performance metrics for comparing the autoencoders during the training process, abnormality detection, and prognostics. The AE convergence is observed through the train and validation set loss on the last training epoch. Abnormality detection capacity is measured by detection coverage, d, and false-positive coverage, f, respectively:

(4) $$d=\frac{n\left(I_{d}^{r}\&\cap I_{d}^{*}\right)}{n\left(I_{d}^{r}\right)}*100$$

(5) $$f=\frac{n\left(I_{n}^{r}\cap I_{d}^{*}\right)}{n\left(I_{n}^{r}\right)}*100$$

The indicator d measures the ratio between the samples correctly signaled by the method as abnormalities and the real set of degradation occurrences, whereas f relates to the ratio of NOC samples highlighted on the same condition and the real entries in the normal state.

Other indicators used in evaluating performance are the discontinuity index $I_{dc}^{\left(j\right)}={\left(N_{int}^{\left(j\right)}\right)}^{-1},$ where $N_{int}^{\left(j\right)}$ is the number of intervals where abnormality was detected, the time interval between the first spotted abnormal point $t_{sp}$, and the concrete tipping point for the degraded stage $t_{d}:\Delta_{usp}=t_{sp}-t_{d}$.

The prognostic capacity, in turn, is quantified by the root-mean-square error (RMSE), NASA’s scoring function adaptation (ns-score) [27,28], and the prognostic horizon. The first two are defined as:

(6) $$ns=\frac{1}{N_{*}}\overset{N_{*}}{\underset{k=1}{\sum }}\exp\left(\beta \left|\Delta^{\left(k\right)}\right|\right)-1$$

(7) $$RMSE=\sqrt{\frac{1}{N_{*}}\overset{N_{*}}{\underset{k=1}{\sum }}{\left(\Delta^{\left(k\right)}\right)}^{2}}$$

where $N_{*}$ indicates the total number of RUL estimations, $\Delta^{\left(k\right)}$ is the difference between the predicted and the real remaining life of the *k*th sample, $\Delta^{\left(k\right)}=r\left(t_{k}\right)-r^{*}\left(t_{k}\right)$, and β is $\frac{1}{14}$ if RUL is underestimated (but $\frac{1}{10}$ otherwise). The ns metric is not symmetric and penalizes overestimation more than underestimation [29].

The prognostic horizon is defined as the time interval between the time $t_{⊤\left(C\right)}$ when a made prediction first meets a specified performance criteria C that continues being satisfied until $t_{eol}$ for all $t^{\left(i\right)}$ such that $t_{⊤\left(C\right)}\leq t^{\left(i\right)}\leq t_{eol}$, thus:

(8) $$H_{⊤\left(C\right)}=t_{eol}-t_{⊤\left(C\right)}$$

Moreover, some metrics proposed by Saxena et al. [27,28] may be used for auxiliary performance inspection, i.e., not designated for a specific finality of tunning or validation in this study within the models’ comparison schema. These metrics are relative accuracy and cumulative relative accuracy.

If relative accuracy (*RA*) is defined as an error measurement in the RUL prediction relative to the actual RUL $r^{*}\left(t_{\lambda }\right)$ at a specified $t_{\lambda }$, then:

(9) $$RA_{\lambda }^{l}=1-\frac{\left|r_{*}^{l}\left(t_{\lambda }\right)-\langle r^{l}\left(t_{\lambda }\right)\rangle \right|}{r_{*}^{l}\left(t_{\lambda }\right)}$$

where l is the index for the *l*th prognostic experiment, $r_{*}^{l}\left(t_{\lambda }\right)$ is the ground truth remaining life at the time $t_{\lambda }$, and $r\left(i_{\lambda }\right)$ is an appropriate central tendency point estimate of the predicted RUL distribution at the time index $t_{\lambda }$.

Since relative accuracy is expressed punctually, to attain an overall view of the algorithm behavior over time, it is necessary to aggregate the measurements as a normalized weighted sum of relative accuracies for all the predictions in one prognosis experiment, resulting in a metric called cumulative relative accuracy, which is:

(10) $$CRA_{\lambda }^{l}=\frac{1}{n\left(p_{\lambda }\right)}\underset{i\in p_{\lambda }}{\sum }w\left(r^{l}\left(t_{\lambda }\right)\right)RA_{\lambda }^{l}$$

where $w\left(r^{l}\left(l\right)\right)$ is a weight factor as a function of RUL at all time instances, $p_{\lambda }$ is the ordered set of all time indexes before $t_{\lambda }$, and $n\left(p_{\lambda }\right)$ is the cardinality of the set $p_{\lambda }$.

Apart from the metrics based on accuracy, it is also important to mention the monotonicity criteria applied as an input of the decision function for the RUL discrimination at the time $t_{i}$, previously elucidated. Lei et al. [13] argue that machinery degradation is an irreversible process and thus should be linked with monotonic increasing or decreasing trends.

There are monotonicity metrics based on the count of finite differences $d/dx=x_{k+1}-x_{k}$ of a health index sequence $X={\left\{x_{k}\right\}}_{k=1:K}$ with $x_{k}$ constituting the value of HI at the time $t_{k}$ [30]. The selected one is described as:

(11) $${Mon}_{1}\left(X\right)=\frac{1}{K-1}|\;No.\;of\;d/dx>0-\;No.\;of\;d/dx<0|$$

where K is the number of the elements of the set X, No.  of   d/d x>0−No.   of   d/d x<0 represents the number of positive and negative differences, respectively, and then ${Mon}_{1}\left(X\right)$ quantifies the absolute difference between them, normalizing it for the interval $\left[0,\;1\right].$

## 3. Results

This chapter presents the results of applying the framework to the CMAPSS database. Section 3.1 describes the CMAPSS dataset, while Section 3.2 shows the results obtained.

### 3.1. Application Example in CMAPSS Dataset

The database chosen for the study is a variant of the Commercial Modular Aero-Propulsion System Simulation (CMAPSS), publicly available and recognized as one of the datasets frequently used for benchmark prediction algorithms. It was recently updated after joint work between NASA and ETH Zurick’s intelligent maintenance systems center, so that the amount of sensor samples has been increased to 1 Hz, making it suitable for the study of the models oriented for large volumes of data.

The CMAPSS-2 [31] is composed of a set of synthetic RTF trajectories, that is, with the artificial degradation of nine turbofan engines that were produced by the simulator from the input of real flight conditions, which are characterized by the scenario descriptor variables: altitude, Mach number, throttle-resolver angle (TRA), and total inlet blade temperature. The base is divided into six units designated for training and another three for testing, with operating conditions slightly different from the others. In this study, only the training data from CMAPSS-2 were used, which does not compromise the feasibility study since the tested model is unsupervised and, therefore, uses only a part of the samples from each unit for training.

**Figure 5:**
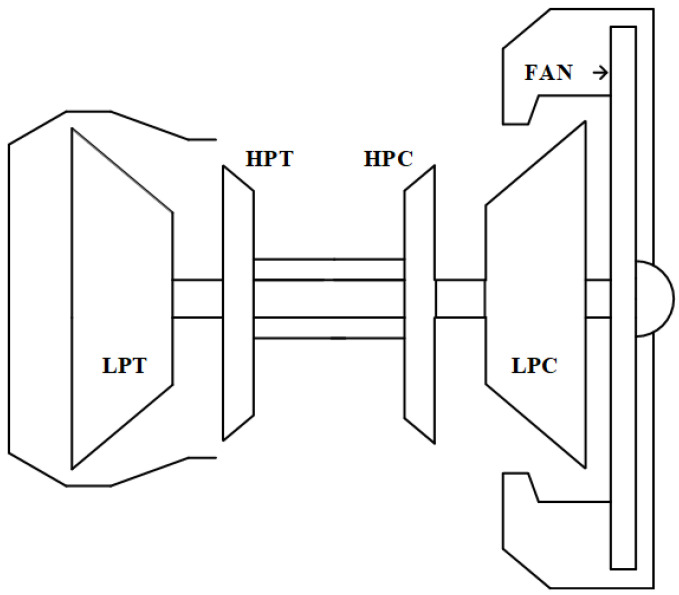
Representation of the main subsystems of the turbofan engine simulated in CMAPSS. From left to right: fan, low-pressure compressor (LPC), high-pressure compressor (HPC), combustion chamber, high-pressure turbine (HPT), and low-pressure turbine (LPT).

The inserted degradation pattern is of a continuous type and is divided into four states: the degradation condition at the beginning of the operation; the normal state; a transition zone between the normal to abnormal conditions; and an abnormal state. The simulation considers the alternating presence of failure modes in the main sub-components of the motor: fan, LPC, HPC, HPT, and LPT. Their deteriorations are modeled by adjustments in flow capacity and efficiency. More information about the modeling can be found in Chao et al.’s work [32]. Figure 5 outlines the allocation of the main subsystems of a turbofan engine.

**Figure 6:**
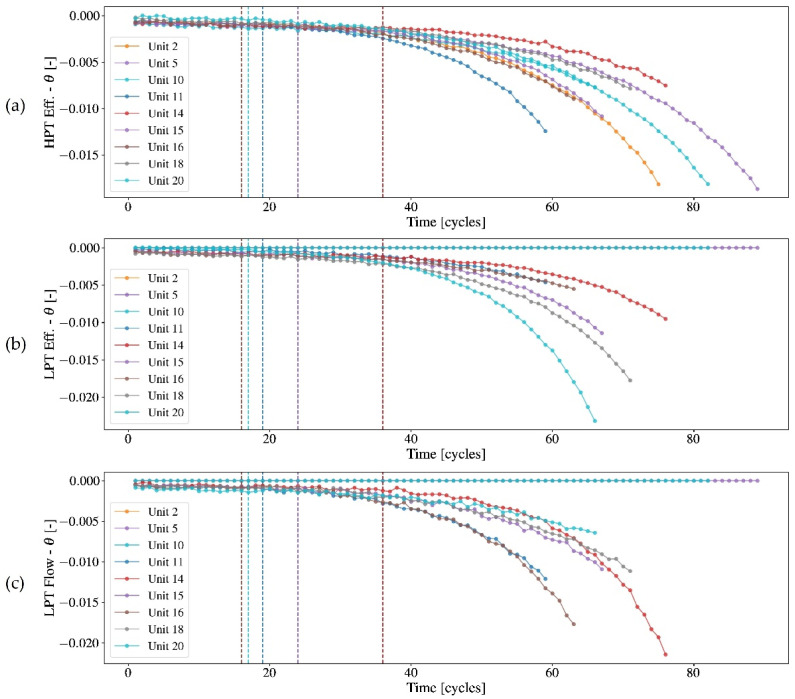
Evolution of flow and efficiency modifier trajectories over time. Degradation is introduced into the system in the timestep indicated by the vertical dashed lines. Therefore, the modulation of these inputs induces a response in the array of sensors. In (**a**) the HPT efficiency modifier is alterated, in (**b**) the LPT effiency modifier and in (**c**) LPT flow modifier.

In this application example, the units have been subjected to high- and low-pressure turbine failure modes with an initial condition of random deterioration of about 10% of the health index implicit in the simulator. Table 3 details the failure modes for each unit and provides additional information on the number of samples, the transition time to abnormality, and end of life ($t_{eol}$) in cycles. Figure 6 details the trajectory imposed on the flow and efficiency modifiers for the tested units.

This application example follows the framework with sets of hyperparameters and fixed neural network architecture, whose feature space is composed of 18 variables, which are the same condition monitoring signals used by Chao et al. [32]. In addition to that, a detailed description of the CMAPSS simulator variables can be found in [31].

The autoencoder models are subject to a validation procedure that consists of two steps: the first one is to evaluate whether its performance (through an analysis of the metrics presented in Section 2.4) surpasses that of a simplified baseline model, which does not use deep learning, and the second one is to compare it with alternatives presented in the literature that employ similar techniques and databases.

The baseline model is built from a simple regression extrapolation procedure of the pre-processed original inputs of the database, following the sequence of steps: down sampling at a rate of 1 sample every 200 (without crossing the limits of operational cycles) and later smoothing by simply moving an average size of 500, so that the samples of this model and the one submitted for validation are similar, and then the application (see Figure 2) of the methodology and performance evaluation, obviously with the metrics of Section 2.4.

### 3.2. Results from Application Example

**Figure 7:**
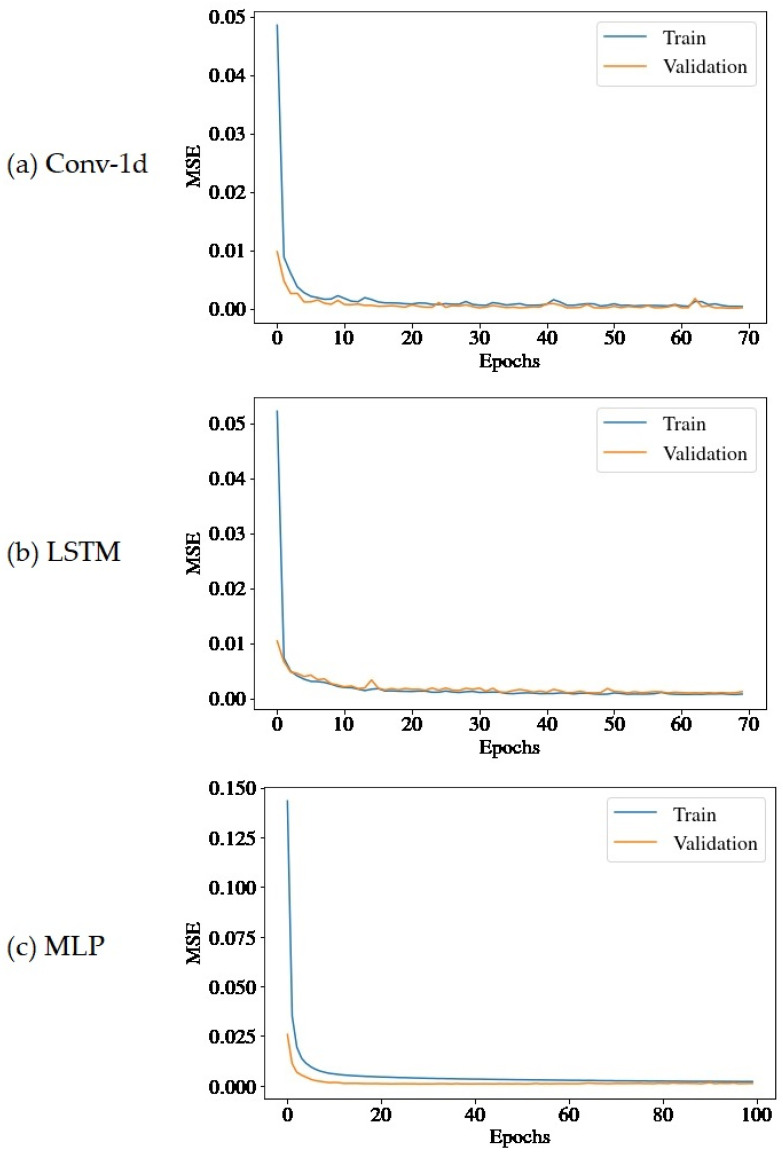
MSE loss convergence during the networks’ training progression: (**a**) Conv-1D; (**b**) LSTM; and (**c**) MLP.

**Figure 8:**
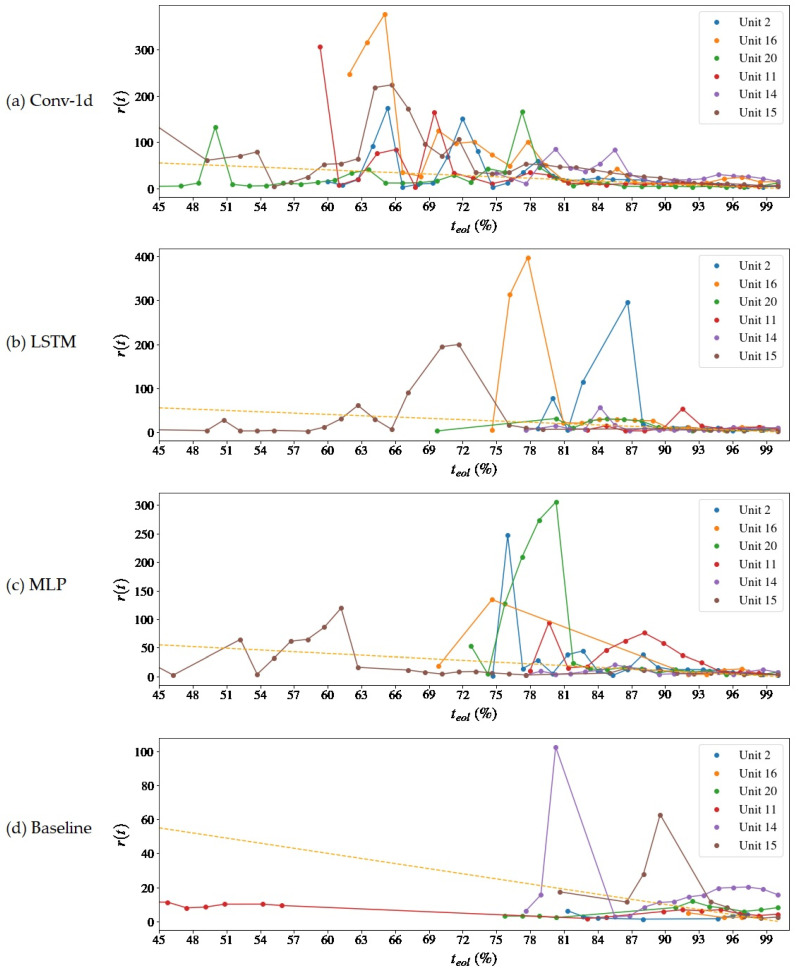
Progression of predictions over units’ operational time—$r\left(t\right)\;x\;t_{i}$ ($t_{eol}$ )—for the autoencoder reconstruction error extrapolation method and Baseline model. The dashed orange line means the ground truth RUL. In (**a**) results are presented for the Conv-1d autoencoder, (**b**) LSTM, (**c**) MLP and (**d**) for the Baseline model.

**Figure 9:**
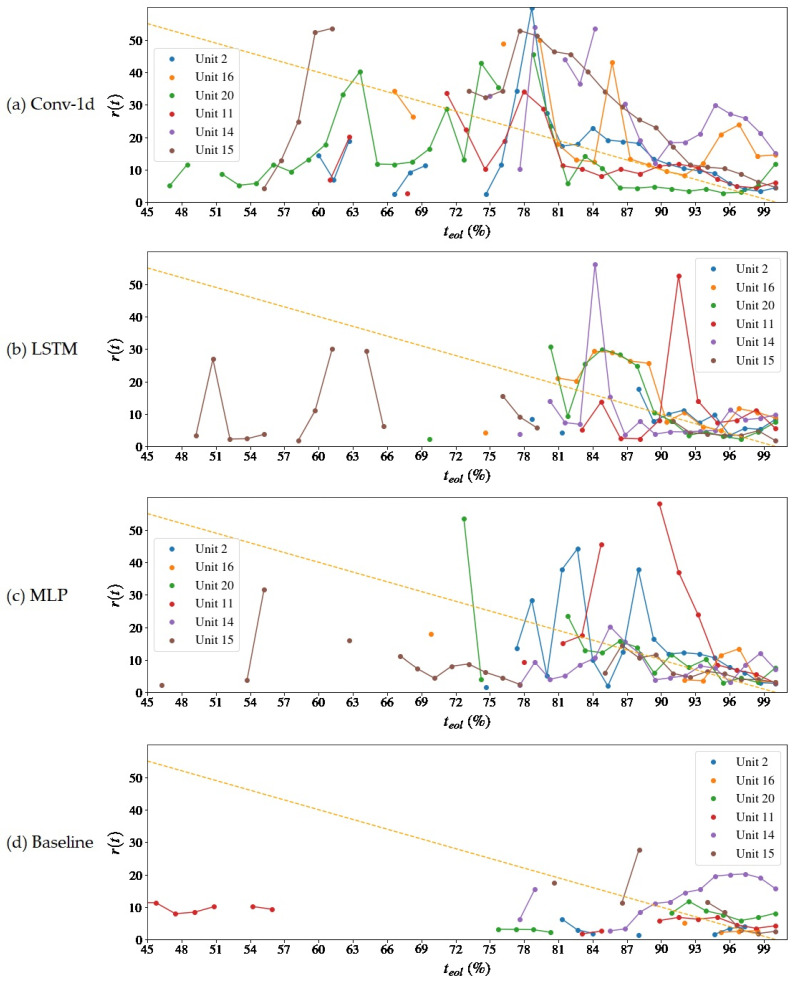
Progression of predictions over units’ operational time—$r\left(t\right)\;x\;t_{i}$ ($t_{eol}$ )—for the autoencoder reconstruction error extrapolation method and Baseline model. Close-up view with error above being 60% $t_{eol}$ suppressed. The dashed orange line means the ground truth RUL. In (**a**) results are presented for the Conv-1d autoencoder, (**b**) LSTM, (**c**) MLP and (**d**) for the Baseline model.

The MSE loss convergence during the networks’ training progression is shown in Figure 7 and the progression of useful life estimations over the course of the operation of the units is presented in Figure 8 and Figure 9. The time instant t, *x*-axis, is normalized in relation to the total life ($t_{eol}$) of the motors and is interpreted as a percentage (0–100%) of $t_{eol}$ or as normalized cycles. The y-axis indicates the predicted RUL at instant *t* (also expressed as a percentage of $t_{eol}$) and the orange dashed line, the real value of the RUL (that is, $t-t_{eol})$ at that instant. It is noted that the beginning of the forecast differs from the units since it is directly related to the abnormality detection capacity, which is made by a criterion similar to that used by Rosa et al. [23], wherein there is a difference in a consecutive set of points of the maximum reconstruction error between the samples in NOC.

For all the analyzed models, the time of the first prediction ($t_{fpt}$) occurred after half of the degradation time of the engines. From 50% to 65% constitutes a region of instability in the forecasts in which there are remaining life estimates that exceed the value of *t_eol_* near 100% or underestimate it close to 1%. This is because the deterioration trends are incipient and have a low rate of change, which makes it difficult for the algorithm to decide which of the curves is the most appropriate, as some have a very similar fit condition. After 70% of the *t_eol_*, a stable convergence zone is formed, and the adherence of the projections to the real RUL curve gradually improves up to 100%, which is the desired behavior. Compared to the baseline model, the proposed models advance to a stable condition much earlier (~65%) than the Baseline (~80%).

From the three models tested, Conv-1d showed the best result in terms of advancing convergence to the actual prognostic result for all units. It can be seen from Figure 8 that it is the model with the most anticipated first average prediction time of all the units and adheres to the reference line of progression of the RUL in about 75% of the *t_eol_*. The MLP model visually manifested a behavior similar to the convolutional one and also presented a zone of forecast instability with high fractional RMSE but with time stamping metrics (*fpt*, H_T(5)_, and H_T(20)_) later compared to the second. The LSTM model did not show a concentrated region of large prediction errors like the previous two, but it did show sparse peaks of high errors for two or three cycles in units 2, 16, 15, and 5. Although it may seem that the LSTM provides more stable predictions, in fact, gaps in the forecasts may occur, especially in the region of 60–75% *t_eol_*, in which large magnitude discrepancies are suppressed by the restriction of the algorithm to disregard RUL estimates, if *r*(*t*) *+ t* exceeds *t_eol_*, above 300 cycles.

For a moving average subsequence of *n* = 500, it can be seen that the three autoencoder models outperform the Baseline, which starts to provide consistent forecasts after 80 normalized cycles have elapsed. An increase in the time window of the moving average could proportionate a positive impact, especially on the base model, as it benefits the most from signal attenuation in regions of instability. However, increasing n reduces the number of samples of each unit available for curve fitting in the prediction algorithm so that the RUL of some units arranged in Table 3 could not be calculated.

**Figure 10:**
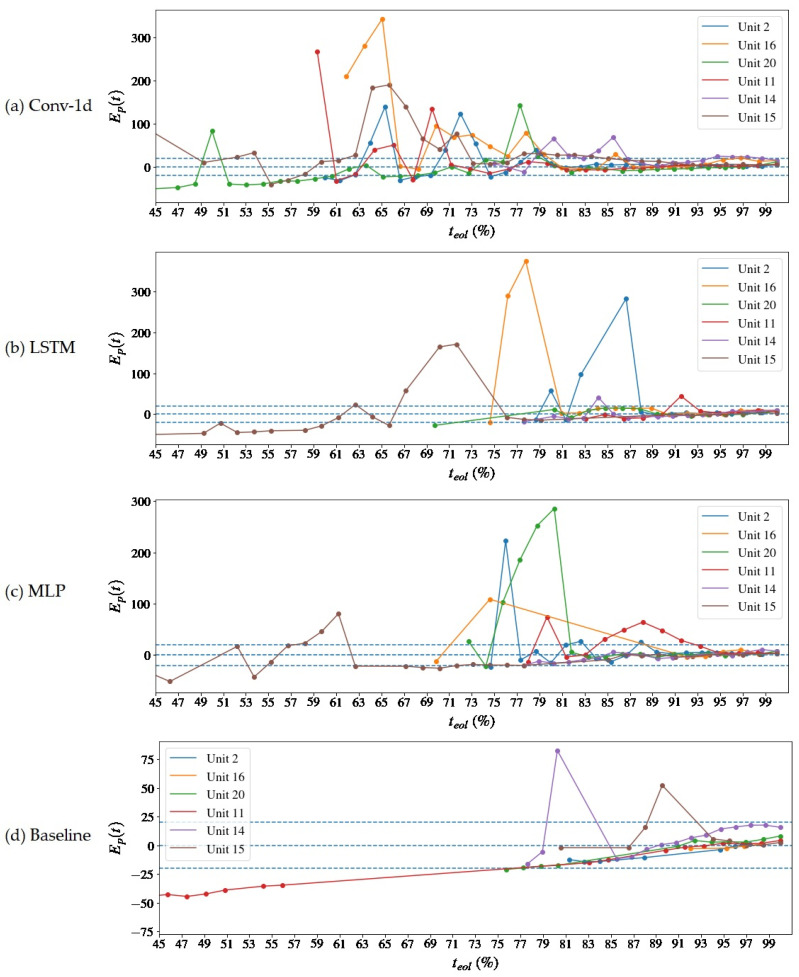
Progression of the prediction error relative to the total life of the asset, $E_{p}=100\times \left(r^{*}\left(t\right)-r\left(t\right)\right)/t_{eol},$ over the time of operation of the units for the reconstruction error extrapolation. The prognostic horizon of 20% is represented by the blue dashed lines. In (**a**) results are presented for the Conv-1d autoencoder, (**b**) LSTM, (**c**) MLP and (**d**) for the Baseline model.

**Figure 11:**
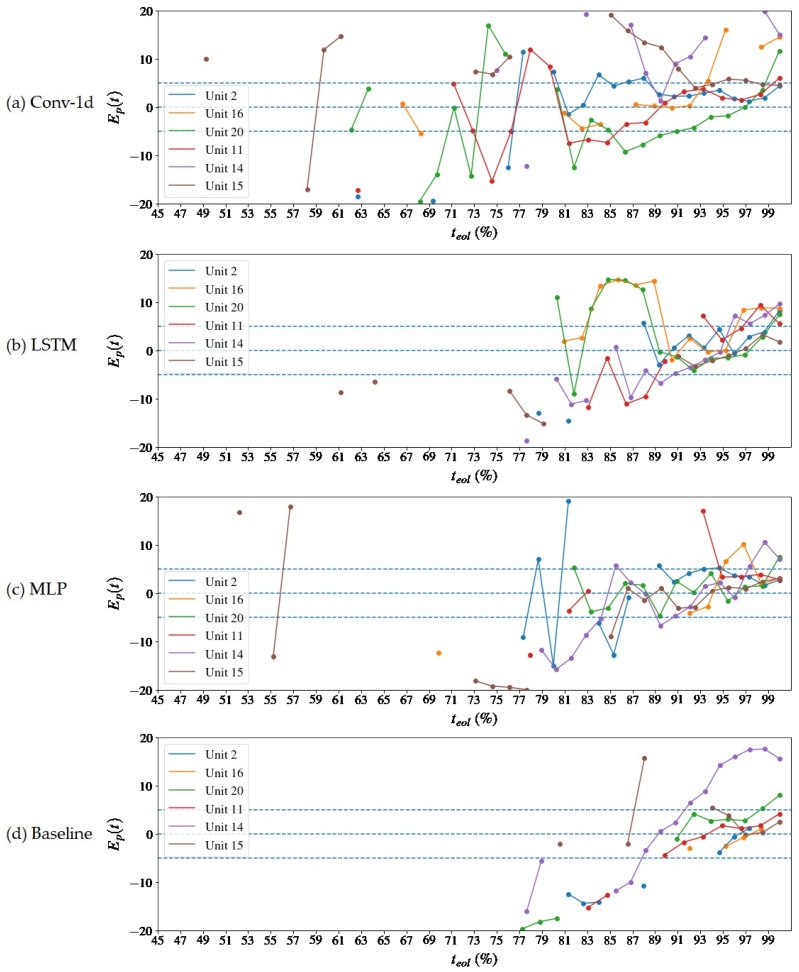
Progression of the prediction error relative to the total life of the asset, $E_{p}=100\times \left(r^{*}\left(t\right)-r\left(t\right)\right)/t_{eol}$, over the time of operation of the units for the reconstruction error extrapolation and baseline methods. The prognostic horizon of 5% is represented by the blue dashed lines. In (**a**) results are presented for the Conv-1d autoencoder, (**b**) LSTM, (**c**) MLP and (**d**) for the Baseline model.

The difference between the forecast and the actual value of the RUL, also expressed as a percentage of the $t_{eol}$, is shown in Figure 10 and Figure 11. The blue dashed lines indicate 20% error limits in Figure 10 and 5% error limits in Figure 11, which is taken as a reference for calculating the prognostic horizon. The proposed method manages to keep the estimates within the error margin of +/− 20% $t_{eol}$, but it has complications in meeting the goal of +/− 5% $t_{eol}$, with only a few units achieving this result even after 80% of the machine’s life. There are two possible reasons for this answer: the first one, mentioned above, is the absence of a global tuning of the model, including the neural architecture, which is not at its optimal performance in terms of training with NOC samples; the second one is the uncertainty regarding the choice of the error threshold for the prognosis, which can increase the estimates above what was expected.

The summary of the results obtained for the values of the performance metrics is presented in Table 4, while Table A1 (in Appendix A), presents all the results organized by unit. Both tables show the RMSE, fractional RMSE’s *L*_1_, *L*_2_, and *L*_3_, time of the first prediction ($t_{fpt}$), the ns (Equation (6)) divided by the total number of estimates, cumulative relative accuracy, and prognostic horizons for 5% and 20% of the errors. It should be noted that *L*_1_, *L*_2_, and *L*_3_ stand for the RMSE fraction only for samples inside the first, second, and third thirds of the second half of the normalized *t_eol_*, respectively.

There is no expressive gain in the RMSE of the proposed model when compared to the Baseline (−15.64%, Table 4) due to the rough projections made at the beginning of the degradation process. When these prognostic samples are disregarded, it is possible to notice a performance gain for this metric, which is expressive from the third third (*L_3_*) and improves the proximity of $t_{eol}$, therefore quantitatively corroborating the notion that the proposed model advances to a state of convergence in the zone before the baseline.

A Table 4 inspection reveals that the global RMSE is lower than the Baseline model. The reasons are that the Baseline model produced less and later estimates in comparison to the autoencoders, as can be viewed in Figure 8 and Figure 9, and when closer to the end of life, the prediction errors tend to be smaller due to the presence of more information about the pronounced degradation. The models start to equate in performance as there is an approximation to the stable convergence zone, and there is a slight divergence between the $RMSE_{L3}$ values. Although the Baseline also has a lower $RMSE_{L3}$, it should be noticed that it has performed fewer predictions even in that region—see Figure 9 and Figure 11. The prognostic horizon is certainly greater for the autoencoder-based solutions, highlighting the Conv-1d, which has the earlier $t_{ftp}$, so a correlation with the $H_{⊤\left(C\right)}$ was already expected. The difference $t_{ftp}$–$H_{⊤\left(C\right)}$ could be interpreted as a latency of the model in the archive or an acceptable error margin.

Moreover, ns, another error evaluation metric, differed from the RMSE’s outcomes by showing a similar quantity overall. This fact is justifiable because, even though the models have differed significantly in global accuracy, all of them displayed a greater tendency to overestimate predictions, which is penalized by this metric. CRA, in turn, follows the RMSE behavior, as they are almost analogous measurements when a linear weighting $w\left(x\right)=x$ (Equation (10)) is taken.

Generally, the proposed autoencoder models are more stable than the Baseline model, detect abnormalities earlier, and enter a region of stable convergence earlier. They manage to meet the margin of error requirement below 20% of $t_{eol}$ for at least a fourth of the unit’s life but struggle to meet the requirement of a 5% forecast horizon.

Finally, the comparison with the literature is based on the publication by Chao et al. [32], who also built deep learning models to estimate the RUL on the CMAPSS-2 basis. This comparison aims to verify if the framework exhibits coherent behavior for the predictions over time. It is made by the qualitative inspection of the prediction errors’ progression, see Figure 10 and Figure 11, which is also plotted by Chao et al. [32] for the same three kinds of layers used in this study. Moreover, some performance measurements taken in this work are compared with the results obtained by the cited author. They are the RMSE and prognostic horizon.

The presented models could not overcome the data-oriented arrangements programmed by Chao et al. [32], nor is this the intention, as they use supervised learning, thus mapping the channel signature throughout the degradation evolution and not only in the NOC. Even if it is not possible to exceed this author in performance, it is important to note that there is a great proximity between the mean squared error values for the stable convergence zone ($RMSE_{L3}$). There is a great similarity between the behavior of the operating time forecasts plotted by this author with the one shown in this work. Greater uncertainty is also demonstrated at the beginning of the forecasting process and gradually reduces until *t_eol_.* It is observed that the use of a supervised technique allows a *t_fpt_* very close to the beginning of the unit’s life and that the supervised method purely derived from ANN can make inferences almost in real time after being trained and generated by a new sample (without the computational costs of the curve fitting). On the other hand, supervised learning techniques tend to be more specific to the application—failure mode—and have a shorter lifespan, requiring retraining to adapt to changes in the operating equipment.

Therefore, the advantage of our approach is its capacity to be easily implemented in an industrial context, which has the particularity of having an abundance of engineering system data in NOC with few recorded faults. Furthermore, real scenarios had lower-quality data labels or unlabeled data. Our framework is designed considering this observation since there is no need to attribute labels or even discriminate sets of abnormal samples. Another point is that we elaborate a complete framework that embraces detection and prognostic models, while Chao et al. [32] focus only on train models for RUL estimation without worrying about scalability. In the end, the proposed framework is more suitable for use in different industrial domains and has an extensive application range because it does not require physical information or intensive knowledge about the fault’s nature and its signature in the sensors’ readings.

## 4. Conclusions

The above results allow us to infer that the framework developed can match the performance with a baseline model that uses simple linear regression on pre-processed signals. It is noteworthy that there is still a large margin of adjustment available through hyperparameter modulations, neural network architectures, and post-processing adjustments for reconstruction errors, among others, to achieve more significant gains.

One future improvement of the prediction algorithm concerns the predictions at the beginning of the degradation process, in which different curves tend to show high $R^{2},$ overestimating the remaining lifetime value. This behavior is somewhat expected, as predictions tend to improve in accuracy as more information about the condition becomes available. However, the decision functions need to be fixed to avoid outliers that exceed $t_{eol}$ values by more than 300% and avoid producing an atypical variability of predictions between two consecutive moments. One way to achieve this is to force downgrade outliers by noting that $dRUL_{real}/dt=-1$. In addition, it is also necessary to calibrate the decision functions better so that the other indicators are taken into account in the hierarchy of functions and sensors, which currently relies heavily on *R*^2^ values.

As for the data pre-processing and reconstruction error post-processing routines, it is feasible to explore new trend-smoothing techniques that do not suppress many samples, which was the case with the moving average used. Applying a moving average of n=2000 points implies losing the equivalent of 30 cycles at the beginning of the abnormality state and contributing to the effect mentioned in the above paragraph. This is a problem for units with low $t_{eol}$, or that operated for a few cycles in an abnormal condition before failure, such as unit 14, as there are few samples designated for the prognostic step. Another alternative would be to merge the labeled inputs with those in normal conditions at an offset of n at most before the detection point.

For future works, a study on the quantification of uncertainties at different stages of the method can be carried out. Potential advances can be made by using Bayesian neural networks or generative models or by adopting probabilistic regressors to extrapolate reconstruction errors. Other sources of uncertainties are in the attribution of the moment of transition to the degraded state, in the attribution of the prognostic trend limit, and, therefore, in the estimation of the RUL, the latter of which absorbs all the variability arising from the decision-making process within the algorithm of prognosis as well as inherits the epistemological and random remnants of the models and data sets, respectively. On the other hand, quantifying multiple sources of uncertainty considerably impacts performance, especially if Monte Carlo random sampling techniques are prevalent. The problem of integrating multiple sources of uncertainty within this framework to produce predictive results with safety margins and fulfill the specificities of the current regulations in a computationally efficient manner is still open to the author. In the absence of the possibility of performing this globally, it is recommended to identify the factors that most contribute to the variability of the prognosis result, such as the definition of the limit error for prognosis, which greatly impacts performance, as observed in the application example.

## Figures and Tables

**Table 1:** Hyperparameter specification of the analyzed autoencoders.

Autoencoder	Hyperparameter	Value/Definition
Conv-1d, LSTM and MLP *	Number of layers—Encoder only	2
	Dropout rate	0.3
	Loss Function	MSE
	Optimization Technique	Adam
	Subsequences Size	200
	Validation data fraction	10%
Conv-1d	Strides	2
	Learning Rate	0.001
	Number of filter units	(32, 16)
	Kernel Size	(15, 10)
	Padding	same
	Activation Function	LeakyReLU
	Epochs	70
LSTM	Activation Function	tanh
	Number of LSTM units	(64, 32)
	Epochs	70
MLP	Activation Function	LeakyReLU
	Neurons	(32, 16)
	Epochs	100

* Here we list the hyperparameters common to the three types of autoencoders.

**Table 2:** Selected model functions $f^{\left(i\right)}\left(c^{\left(i\right)},x\right)$ that cause the vector *f* subject to be minimized in accordance with the algorithm presented in Figure 2.

$\mathrm{Function}\;f^{\left(i\right)}$	Behavior
1	$c_{1}x+c_{0}$
2	$c_{2}x^{2}+c_{1}x+c_{0}$
3	$c_{1}log\left(x\right)+c_{0}$
4	$c_{2}e^{c_{1}x}+c_{0}$

**Table 3:** Information about subset samples of each unit (adapted from [32]).

Dataset Fraction	Unit (u)	Rows (10^4^)	$t_{s}$ * (Cycles)	*t_eol_* (Cycles)	Failure Mode
Training	2	8.5	17	75	HPT
	5	10.3	17	89	HPT
	10	9.5	17	82	HPT
	16	7.7	16	63	HPT + LPT
	18	8.9	17	71	HPT + LPT
	20	7.7	17	66	HPT + LPT
Test	11	6.6	19	59	HPT + LPT
	14	1.6	36	76	HPT + LPT
	15	4.3	24	67	HPT + LPT

* *t_s_* represents the real transition time between normal and degraded conditions.

**Table 4:** Mean (μ) and standard deviation (σ) of the performance metrics computed for each model and the autoencoders overall.

Performance Metrics	Conv-1d		MLP		LSTM		Conv-1d, MLP, and LSTM Overall		Baseline	
	µ	σ	µ	σ	µ	σ	µ	σ	µ	σ
*RMSE*	49.702	23.942	44.116	30.256	42.505	36.587	45.441	6.322	14.702	12.102
*RMSE_L1_*	122.926	81.626	44.815	5.514	49.569	17.053	72.437	41.020	52.499	0.000 *
*RMSE_L2_*	35.958	15.820	63.927	52.562	52.505	66.543	50.797	26.198	20.968	15.921
*RMSE_L3_*	8.668	7.929	9.312	9.856	18.267	27.008	12.082	10.503	7.964	5.549
*t_fpt_*	57.627	11.532	65.039	15.695	62.454	19.762	61.707	4.115	75.633	19.729
*ns*	0.403	0.002	0.402	0.002	0.400	0.002	0.402	0.000	0.400	0.002
*CRA*	−0.311	0.912	−0.488	0.708	−0.482	0.785	−0.427	0.103	0.006	0.633
*H_T(5)_*	11.767	6.123	8.205	7.847	4.169	4.690	8.047	1.581	4.724	2.984
*H_T(20)_*	20.694	5.336	14.735	4.750	12.072	8.399	15.834	1.959	6.643	4.270

* Standard deviation value is 0 because there was just one sample.

## Data Availability

Publicly available datasets were analyzed in this study. These data can be found here: https://www.nasa.gov/content/prognostics-center-of-excellence-data-set-repository (accessed on 4 December 2022).

## Appendix A

Table A1 presents the performance metrics for each unit’s remaining life evaluation of the tested models.

**Table A1:** Performance metrics for each unit’s remaining life evaluation of the tested models. *L*_1_, *L*_2_, and *L*_3_ stand for the RMSE fraction only for samples inside the first, second, and third thirds of the normalized *t_eol_* second half, respectively.

Autoencoder	Unit	*RMSE*	*RMSE*_*L*1_	*RMSE*_*L*2_	*RMSE*_*L*3_	*t_fpt_*	*ns*	*CRA*	*H*_*T(*5*)*_	*H*_*T(*20*)*_
Conv-1d	2	28.174	12.882	14.076	8.793	61.333	0.402	−0.052	4	9.333
	5	39.661	9.798	7.56	1.299	64.045	0.403	0.408	12.36	33.708
	10	11.883	8.405	8.76	2.491	57.317	0.405	0.5	9.756	42.683
	11	42.548	5.922	5.427	6.443	59.322	0.402	0.095	1.695	30.508
	14	91.845	91.845	35.161	48.548	97.368	0.407	−31.796	0	0
	15	70.579	37.695	21.768	14.243	52.239	0.404	−0.791	0	19.403
	16	82.639	83.927	6.662	3.703	47.619	0.404	−0.417	11.111	23.81
	18	58.992	10.021	5.521	2.912	30.985	0.4	0.26	32.394	32.394
	20	24.333	16.163	8.475	3.301	31.818	0.398	0.43	15.152	51.515
MLP	2	28.174	12.882	14.076	8.793	61.333	0.402	−0.052	4	9.333
	5	39.661	9.798	7.56	1.299	64.045	0.403	0.408	12.36	33.708
	10	11.883	8.405	8.76	2.491	57.317	0.405	0.5	9.756	42.683
	11	42.548	6.922	5.427	6.443	59.322	0.402	0.095	1.695	30.508
	14	91.845	91.845	35.161	48.548	97.368	0.407	−31.796	0	0
	15	70.579	37.695	21.768	14.243	52.239	0.404	−0.791	0	19.403
	16	82.639	83.927	6.662	3.703	47.619	0.404	−0.417	11.111	23.81
	18	58.992	10.021	5.521	2.912	30.986	0.4	0.26	32.394	32.394
	20	24.333	16.163	8.475	3.301	31.818	0.398	0.43	15.152	51.515
LSTM	2	28.174	12.882	14.076	8.793	61.333	0.402	−0.052	4	9.333
	5	39.661	9.798	7.56	1.299	64.045	0.403	0.408	12.36	33.708
	10	11.883	8.405	8.76	2.491	57.317	0.405	0.5	9.756	42.683
	11	42.548	6.922	5.427	6.443	59.322	0.402	0.095	1.695	30.508
	14	91.845	91.845	35.161	48.548	97.368	0.407	−31.796	0	0
	15	70.579	37.695	21.768	14.243	52.239	0.404	−0.791	0	19.403
	16	82.639	83.927	6.662	3.703	47.619	0.404	−0.417	11.111	23.81
	18	58.992	10.021	5.521	2.912	30.986	0.4	0.26	32.394	32.394
	20	24.333	16.163	8.475	3.301	31.818	0.398	0.43	15.152	51.515
Baseline	2	28.174	12.882	14.076	8.793	61.333	0.402	−0.052	4	9.333
	5	39.661	9.798	7.56	1.299	64.045	0.403	0.408	12.36	33.708
	10	11.883	8.405	8.76	2.491	57.317	0.405	0.5	9.756	42.683
	11	42.548	6.922	5.427	6.443	59.322	0.402	0.095	1.695	30.508
	14	91.845	91.845	35.161	48.548	97.368	0.407	−31.796	0	0
	15	70.579	37.695	21.768	14.243	52.239	0.404	−0.791	0	19.403
	16	82.639	83.927	6.662	3.703	47.619	0.404	−0.417	11.111	23.81
	18	58.992	10.021	5.521	2.912	30.986	0.4	0.26	32.394	32.394
	20	24.333	16.163	8.475	3.301	31.818	0.398	0.43	15.152	51.515
